# Supplementary material for: Exploring the role of the UK renal social worker: The nexus between health and social care for renal patients
Source: PLoS One. 2022 Sep 26;17(9):e0275007. doi: 10.1371/journal.pone.0275007 (PMC9512173; doi:10.1371/journal.pone.0275007)
Supplement: Attachment — Submitted filename: Response to reviewers_07.09.2022.docx [file pone.0275007.s004.docx]

**Journal requirements:**

1. Please review your reference list to ensure that it is complete and correct. If you have cited papers that have been retracted, please include the rationale for doing so in the manuscript text, or remove these references and replace them with relevant current references. Any changes to the reference list should be mentioned in the rebuttal letter that accompanies your revised manuscript. If you need to cite a retracted article, indicate the article’s retracted status in the References list and also include a citation and full reference for the retraction notice.

The listed references have remained the same, no changes have been made.

**Reviewer comments:**

*Thank you for responding thoroughly to each comment in my previous review. I still have a minor suggestions to make. Secondary data is mentioned under Methods page 5 but explained on page 8 line 168, I suggest it is explained under Methods. Secondary data: This is also written in the abstract without any explanation, perhaps it should be explained there or deleted?*

We thank the reviewer for their second, thorough assessment of our manuscript. We hope that these changes satisfactorily address the minor comments provided.

The mention of secondary data has been removed on page 5, so now the sentence only explains the difference in the aims of study part 1 and study part 2, as follows:

*The first part involved an exploration of the adult RSW role; the second part consisted of an uncontrolled, pre-post evaluation of RSW involvement.*

Secondary data are explained in detail under the section *methods and data collection tools*.

In the abstract, an explanation has been added to clarify that secondary data includes data on caseloads and audit files where available.
